# CBCT Assessment of Posterior‑Mandibular Bone Density in Well‑Controlled Type 2 Diabetes: An Analytical Cross‑Sectional Study

**DOI:** 10.21142/2523-2754-1403-2026-298

**Published:** 2026-07-10

**Authors:** Peyman Zamanipour, Nava Naghibi, Najmeh Anbiaee, Ershad Aghasizadeh, Arsalan Shahri

**Affiliations:** 1 Department of Orthodontics, School of Dentistry, Mashhad University of Medical Sciences. Mashhad, Iran. zmppeyman@gmail.com Mashhad University of Medical Sciences Department of Orthodontics, School of Dentistry Mashhad University of Medical Sciences Mashhad Iran zmppeyman@gmail.com; 2 Department of Periodontology, School of Dentistry, Mashhad University of Medical Sciences. Mashhad, Iran. NaghibiN@mums.ac.ir, aghaszadehe@mums.ac.ir Mashhad University of Medical Sciences Mashhad University of Medical Sciences Mashhad Iran NaghibiN@mums.ac.ir aghaszadehe@mums.ac.ir; 3 Oral and Maxillofacial Radiology Department, School of Dentistry, Mashhad University of Medical Sciences. Mashhad, Iran. anbiaeen@mums.ac.ir Mashhad University of Medical Sciences Oral and Maxillofacial Radiology Department, School of Dentistry Mashhad University of Medical Sciences Mashhad Iran anbiaeen@mums.ac.ir; 4 Dental Materials Research Center, Mashhad Dental School, Mashhad University of Medical Sciences. Mashhad, Iran Mashhad University of Medical Sciences Dental Materials Research Center, Mashhad Dental School Mashhad University of Medical Sciences Mashhad Iran

**Keywords:** Bone Density, Cone-Beam Computed Tomography, Diabetes Mellitus, densidad ósea, tomografía computarizada de haz cónico, diabetes mellitus

## Abstract

**Objective:**

Implant success hinges on recipient‑site bone quality, which systemic diseases like diabetes may impair. Diabetes alters bone metabolism, lowering bone mineral density, promoting osteopenia, and elevating fracture risk, potentially jeopardizing osseointegration. Using cone‑beam computed tomography (CBCT), this study assessed how well‑controlled diabetes influences radiographic bone density in the posterior mandible.

**Materials and Methods:**

Cone‑beam CT scans from 48 adults scheduled for posterior mandibular implants were retrospectively analyzed. Cancellous bone density at the planned osteotomy sites was measured in Hounsfield units (HU) with Romexis software. Patients were divided into well‑controlled diabetics (HbA1c 6.1-8%, n = 24) and non‑diabetic controls (n = 24). Mean HU values were analysed with independent‑samples t‑tests for diabetes status, sex, and dichotomised age (56 or less vs more than 56 years). Significance was set at p < 0.05.

**Results:**

The cohort comprised 26 men (54.2 %) and 22 women (45.8 %) with a mean age of 56.3 years. Mean cancellous density did not differ between diabetic and control groups (p = 0.199) and was independent of sex (p = 0.207) and age (p = 0.511).

**Conclusion:**

With good glycemic control, diabetic patients show the same cancellous bone density in the posterior mandible as non‑diabetic patients, indicating that effective diabetes management prevents trabecular bone changes at planned implant sites.

## Resumen

**Objetivo:**

El éxito de los implantes depende de la calidad ósea del sitio receptor, la cual puede verse afectada por enfermedades sistémicas como la diabetes. La diabetes altera el metabolismo óseo, reduce la densidad mineral ósea, favorece la osteopenia y aumenta el riesgo de fracturas, lo que podría comprometer la osteointegración. Mediante tomografía computarizada de haz cónico (CBCT), este estudio evaluó cómo la diabetes bien controlada influye en la densidad ósea radiográfica en la mandíbula posterior.

**Materiales y métodos:**

Se analizaron retrospectivamente tomografías computarizadas de haz cónico de 48 adultos programados para colocación de implantes en la región posterior de la mandíbula. La densidad del hueso esponjoso en los sitios planificados de osteotomía se midió en unidades Hounsfield (UH) utilizando el software Romexis. Los pacientes se dividieron en diabéticos bien controlados (HbA1c 6,1-8 %, n = 24) y controles no diabéticos (n = 24). Los valores medios de UH se analizaron mediante pruebas t de muestras independientes según la condición de diabetes, el sexo y la edad dicotomizada (≤56 frente a >56 años). El nivel de significancia se fijó en p < 0,05.

**Resultados:**

La cohorte estuvo conformada por 26 hombres (54,2 %) y 22 mujeres (45,8 %), con una edad media de 56,3 años. La densidad media del hueso esponjoso no mostró diferencias significativas entre el grupo diabético y el grupo control (p = 0,199), y fue independiente del sexo (p = 0,207) y la edad (p = 0,511).

**Conclusión:**

En presencia de un buen control glucémico, los pacientes diabéticos presentan la misma densidad de hueso esponjoso en la mandíbula posterior que los pacientes no diabéticos, lo que indica que un manejo eficaz de la diabetes previene cambios trabeculares en los sitios planificados de colocación de implantes.

## INTRODUCTION

Dental implants now achieve survival rates near 95 %, making them a mainstream option for oral rehabilitation^.(^^1^^-^^4^ Their long term success, however, is affected by both implant related, surgical-related, and patient related factors, including the quantity and quality of recipient bone as well as biographical characteristics such as age and gender and the presence of systemic diseases.^2^^,^^5^^-^^7^^)^ Adequate bone density underlies primary stability, defined as the absence of micromotion at the implant-bone interface immediately after placement, and a strong positive association exists between primary stability and long term clinical outcomes.^2^^,^^3^^)^ Once primary stability is secured, predictable osseointegration can follow; this is the direct functional and structural bond between living, organised bone and the surface of a load bearing implant.^8^^,^^9^

Systemic conditions such as diabetes mellitus can undermine implant therapy by altering bone metabolism,^10^^)^ manifesting as reduced bone mineral density (BMD) and osteopenia.^8^^,^^11^^)^ Diabetes is among the most prevalent and costly chronic diseases, with projections exceeding 642 million affected worldwide by 2040.^12^^)^ Its complications: retinopathy, nephropathy, neuropathy, macrovascular disease, and impaired wound healing, combined with compromised neutrophil function and delayed tissue repair, predispose patients to periodontal disease, abscesses, and implant failure.^13^^)^ Studies of diabetes and skeletal BMD have yielded mixed results: some report significantly lower BMD in diabetic versus non-diabetic cohorts,^10^^,^^13^^-^^15^ whereas others find no such difference.^11^^,^^16^

Most earlier studies assessed bone mineral density with two‑dimensional methods.^10^^,^^13^^-^^16^^)^ Dual‑energy X‑ray absorptiometry (DEXA) is still the clinical gold standard,^16^ yet cone‑beam computed tomography (CBCT) correlates strongly with DEXA values.^14^^,^^17^^,^^18^ Introduced to dentistry in 1998, CBCT provides three‑dimensional, cross‑sectional images that permit site‑specific evaluation of jaw BMD and bone dimensions.^3^^,^^13^

Previous research on diabetes and jaw‑bone quality is limited by two‑dimensional analysis, heterogeneous glycemic control, and failure to consider age and gender as potential confounding factor. Thus, the present study tested the hypothesis that diabetes status, age, and gender do not significantly affect three‑dimensional radiographic density of posterior‑mandibular basal bone in a well-controlled diabetic patient as measured by CBCT.

## MATERIAL AND METHODS

### Study design

This analytical cross‑sectional study was performed at the Faculty of Dentistry, Mashhad University of Medical Sciences (MUMS), Mashhad, Iran, from December 2022 to August 2023. All cone‑beam computed tomography (CBCT) scans were originally obtained for routine clinical care and were retrospectively retrieved from the records of a private dental radiology clinic located in Mashhad, Iran. The study protocol was approved by the Research and Ethics Committee of Mashhad University of Medical Sciences (IR.MUMS.DENTISTRY.REC.1401.104; 12 December 2022). Acting on the waiver criteria-minimal risk, impracticability of seeking consent, important social value, and use of fully anonymised existing data-set out in CIOMS Guideline 10, U.S. 45 CFR 46.116, and reaffirmed in the World Medical Association’s 2024 Declaration of Helsinki, the committee waived the requirement for individual informed consent because the research used retrospectively collected, fully anonymised CBCT datasets.

### Sample‑size calculation

Using a 95 % confidence level and 80 % power, and applying parameters reported by Rai et al.,^15^^)^ the required sample size per group was calculated with the two‑independent‑sample means formula as 24 participants.

### Participants and eligibility criteria

Electronic records from 2018 - 2023 were screened in the Implant Department of the Faculty of Dentistry and several private periodontal clinics in Mashhad, yielding 48 edentulous adults scheduled for posterior‑mandibular implants who satisfied the inclusion criteria; based on documented medical histories they were assigned to a well‑controlled diabetes group (Hemoglobin A1c 6.1 - 8 %; n = 24) or to non‑diabetic controls (n = 24), and subsequent checks confirmed no significant age or sex differences between the groups.

### Inclusion criteria

• High quality CBCT of an edentulous posterior mandible (≥ 1 year post extraction).
• Basal cancellous region distal to the mental foramen available for measurement.
• A confirmed diagnosis of diabetes mellitus for the diabetic group, with controlled diabetes defined as a Hemoglobin A1c (HbA1c) level between 6.1% and 8%.^(19)^

### Exclusion criteria

• Motion/metal artefacts in the region of interest.
• Pathology (cyst, tumour), metabolic bone disease (osteoporosis, osteopetrosis, osteomyelitis), prior mandibular surgery, or maxillofacial trauma.
• Systemic disease or infection other than diabetes or medication affecting bone metabolism.

### CBCT acquisition and processing

All CBCT scans were acquired with a Planmeca ProMax 3D Mid unit (Planmeca, Helsinki, Finland) using a 10 × 10 cm field of view at the system’s normal‑resolution setting. Exposure parameters were individually adjusted to suit each patient’s anatomy and diagnostic requirements. The Digital Imaging and Communications in Medicine (DICOM) files were then imported into Planmeca Romexis software, version 6.0.0.3 (Planmeca, Finland). Cancellous‑bone density in the basal portion of the posterior mandible was analysed at a voxel size of 200 µm. All measurements were performed in a dimly lit room on a 15‑inch monitor (1366 × 768 pixels, Windows 10).

### Bone density measurement

**Figure 1:**
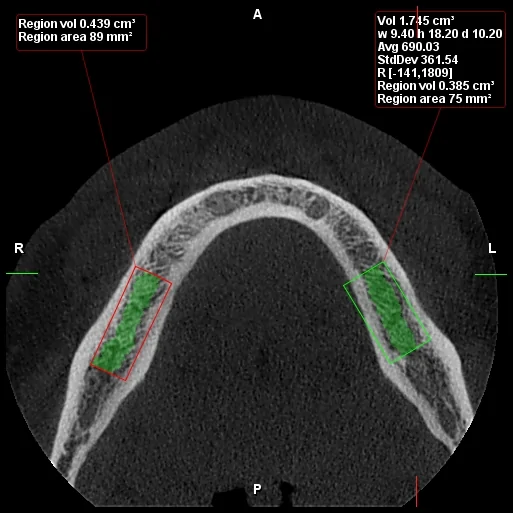
Bone mineral density measurement in the axial view.

A trained dentist extracted grey‑scale values, expressed as Hounsfield‑unit equivalents (HU), from regions at least one year post‑extraction. For each case, a cubic volume of ≥ 0.1 cm³ distal to the mental foramen was selected. Cross‑sectional images were first reformatted along a customised panoramic curve at 1‑mm intervals; in every slice, at least 1 mm was left intact above the superior border of the inferior alveolar canal, and cortical bone or residual roots were excluded (Figures 1and2).

In bilaterally edentulous patients, the mean HU of both sides represented the overall basal density, while side‑specific values were retained for later comparison. In unilaterally edentulous patients, the HU of the single edentulous side served both as the individual’s overall density and as the side‑specific value for group analyses. Mean densities for the diabetic and non‑diabetic groups were ultimately compared, after confirming that age and sex were evenly distributed between groups to minimise confounding.

**Figure 2:**
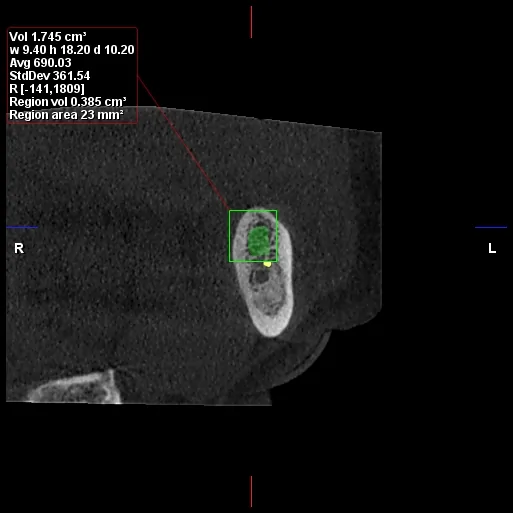
Bone mineral density measurement in the coronal view.

### Observer reliability

After two weeks, ten randomly chosen scans were remeasured. In terms of acceptable agreement, intra-observer intraclass correlation coefficients were higher than [ICC ≥ 0.80]. 

### Statistical analysis

Normality of data were confirmed using Shapiro-Wilk test, after which independent‑samples t‑tests compared mean HU values by diabetes status, sex, age group (≤ 56 y vs > 56 y), and mandible side. Results are expressed as mean ± SD (95 % CI); p < 0.05 was considered significant. Analyses were run in SPSS v26 (IBM, Armonk, NY, USA).

## RESULTS

Forty‑eight posterior‑mandibular CBCT scans were analyzed (26 men [54.2 %] and 22 women [45.8 %]; mean age 56.31 ± 8.0 years, range 40 - 72). The diabetic (n = 24) and non‑diabetic (n = 24) cohorts were matched for sex (13 men and 11 women each) and age (56.29 ± 8.2 vs 56.33 ± 7.9 years). 

Bone‑density data are summarized in Table 1. Mean cancellous BMD was lower in diabetics than in controls (611.83 ± 180.99 HU vs 676.83 ± 163.94 HU), but the difference was not statistically significant (p = 0.199). Right‑ and left‑side analyses likewise showed no significant group differences (p = 0.786 and 0.533, respectively). Although men displayed higher HU values than women on both sides and overall, none of these contrasts reached significance (overall p = 0.207). Basal density was also comparable between patients ≤ 56 years and those > 56 years (overall p = 0.511; right p = 0.756; left p = 0.260). 

**Table 1:** The mean ± standard deviation (SD) of bone mineral density (BMD) in terms of Hounsfield unit (HU) for each group according to diabetes status, gender, and age

		Diabetes status		Gender			Age (years)	
		Diabetic	Non-diabetic	Male	Female	≤ 56	> 56
Right side	N	17	21	23	15	20	18
	Mean ± SD	641.1 ± 194.2	657.4 ± 173.7	689.3 ± 207.1	589.9 ± 112.4	641.1 ± 146.1	660.1 ± 216.9
	P value	0.786	0.098	0.756			
Left side	N	19	16	21	14	19	16
	Mean ± SD	616.1 ± 193.1	660.1 ± 219.8	642.5 ± 226.6	626.6 ± 171.8	672.2 ± 197.4	593.3 ± 209.4
	P value	0.533	0.825	0.260			
Total	N	24	24	26	22	25	23
	Mean ± SD	611.8 ± 180.9	676.8 ± 163.9	673.7 ± 195.2	609.6 ± 141.3	660.3 ± 161.5	626.8 ± 188.6
	P value	0.199	0.207	0.511			

## DISCUSSION

The present study suggests that BMD in the diabetic group was lower than that in the non-diabetic group; however, the difference was not statistically significant. These findings are consistent with that of Patil et al. ^16^ who investigated bone density among individuals with diabetes, controlled diabetics, and healthy subjects. Their study found no significant differences between the three groups. Likewise, Kayipmaz et al. ^11^ revealed that there was no statistically significant variation in the posterior mandibular bone density. An investigation by Jolly et al.,^20^^)^ revealed no significant difference in bone density between diabetic and well-controlled patients.

However, the results of the present investigation do not support some of the previous studies. Nemtoi et al. ^13^^)^ used CBCT to evaluate bone density in the posterior mandible. According to their findings, people with diabetes showed a statistically significant decrease in bone density. This inconsistency may be due to two-dimensional and qualitative assessment of BMD. Furthermore, matching the two groups by gender and age was not mentioned. Shalu Rai et al. ^15^ showed a high level of concordance between diabetes status and BMD in cortical and cancellous bones. This discrepancy could be attributed to the absence of HbA1c upper limit for the inclusion criteria. Moreover, measurements of bone density were made in the maxilla and anterior jaw regions in addition to the posterior mandible. 

Other researchers used the HbA1C level to categorize the diabetic group. In a study by Zhang et al., ^(^^10^^)^ results demonstrated a significant inverse correlation between posterior mandibular bone density and three-month average blood glucose levels (HbA1c). Likewise, El Saadawy et al. ^14^ found a significant correlation between HbA1c and bone density. Although, the results may be more accurate and possibly different if the diabetic group is divided according to the degree of disease control. However, a possible explanation for these results may also be due to the smaller size of the sample and two-dimensional BMD evaluation.

In addition, these relationships may partly be explained by molecular changes occur in cells of diabetic patients. Insulin regulates normal skeletal growth in addition to being a crucial hormone for glucose regulation. This hormone promotes bone matrix synthesis but does not control bone resorption. Insulin has both direct and indirect effects on bone metabolism. Directly, it stimulates osteoblastic matrix synthesis, and indirectly, it promotes the hepatic production of insulin-like growth factor I (IGF-I), which enhances matrix synthesis.^8^^)^

Based on gender-wise assessment in this investigation, the mean bone density in women was lower than in men, but this difference was also not significant. In the study conducted by Rai et al.,^15^^)^ it was observed that cortical and cancellous bone density was higher in men. They evaluated all areas of the jaws and included a wider age range (31 to above 70 years), but the findings imply that bone density is influenced by a number of other factors besides gender. Likewise, Lee et al. ^21^ reported that trabecular bone density was higher in men than in women. In women, estrogen accumulation during the premenopausal years contributes to increased bone density. However, the loss of estrogen's protective effects during the postmenopausal phase results in decreased bone density and an increased risk of osteoporosis. In men, a progressive decrease in steroid hormone levels has been noted between the ages of 30 and 40, which may contribute to reduced bone density during this period. However, the rate of hormonal decline tends to become slower after this age range.^22^^)^ Also, it may be due to higher occlusal force in men.^23^

Another finding is that bone density did not change significantly with age. In addition, according to a study by Rai et al. ^15^^)^ age-wise assessment of BMD showed no statistically significant change. In contrast to these findings, Ono et al. ^24^ found the highest BMD value between the 25-49 years old in both male and female. In male, 9.8% decrease in BMD was observed until 74 years. After that, there were no more alterations observed. Females showed a declining trend over the course of their lives, with values as low as 76% of the initial peak value. The observed decrease in BMD could be attributed to hormonal changes and nutritional insufficiency.^25^

The imaging modality used to evaluate the jaws precisely is cone-beam computed tomography (CBCT).^26^^)^ CBCT offers several advantages, including ease of imaging, high image accuracy, reduced artifacts, lower radiation dose compared to conventional CT, faster scan times, and cost-effectiveness. According to studies, measurements obtained from CBCT are significantly more accurate than those from panoramic imaging.^27^^)^ The Hounsfield Unit (HU) is a suitable index for quantitatively measuring bone mineral density. Although, this index commonly used in computed tomography (CT imaging), but A strong correlation between the HUs of CT scan and the gray scales of CBCT was found.^28^^,^^29^

This study focused on assessing the density of basal cancellous bone in the posterior region of the mandible because the decrease in the number of bony trabeculae is less noticeable in the alveolar process of the jaw; this may be due to the mechanical forces exerted by the teeth.^30^^)^ In a study conducted by Prozorova et al.,^31^^)^ bone density was assessed using CBCT in patients with diabetes mellitus. The results showed a notable decrease in bone density at the level of the cemento-enamel junction (CEJ) in diabetic individuals. Changes were less pronounced in the middle third, while a slight increase in bone density was observed in the apical region. These inconsistencies can be attributed to the measurement of bone density in the alveolar bone. However, Duncea et al. ^5^^)^ discovered associations between the BMD of the mandible and lumbar spine, femoral neck, and total hip BMD, indicating that osteoporosis can impact on mandibular BMD.

Additional studies evaluating BMD in other bones than the jaw will be needed to develop a complete picture of BMD in diabetic patients. Also, further investigations are required to examine the impact of the length of time that diabetes has been present since the onset of the disease.

## CONCLUSION

These findings suggest that in patients with well-controlled diabetes, age, gender, and diabetic status do not significantly affect mandibular bone density, supporting that diabetes under optimal control may only be considered a relative contraindication rather than an absolute one for implant therapy.
